# Effect of Age at First Calving on the Reproduction Parameters, Metabolic Profile, and Fatty Acid Composition of Polish Holstein Friesian (PHF) and Crossbreds PHF × Swedish Red (SRB) Cattle

**DOI:** 10.3390/metabo14110583

**Published:** 2024-10-27

**Authors:** Paweł Solarczyk, Marcin Gołębiewski, Jan Slósarz, Antonio Natalello, Martino Musati, Ruggero Menci, Tomasz Sakowski, Karol Tucki, Kamila Puppel

**Affiliations:** 1 Department of Animal Breeding, Institute of Animal Sciences, Warsaw University of Life Sciences, Ciszewskiego 8, 02-786 Warsaw, Poland; 2 Department of Agriculture, Food and Environment, University of Catania, Via Santa Sofia 100, 95123 Catania, Italy; 3 FiBL France, Research Institute of Organic Agriculture, Pôle Bio 150, Avenue de Judée, 26400 Eurre, France; 4 Department of Biotechnology and Nutrigenomics, Institute of Genetics and Animal Biotechnology, Polish Academy of Sciences, Postępu 36A, 05-552 Jastrzębiec, Poland; 5 Department of Production Engineering, Institute of Mechanical Engineering, Warsaw University of Life Sciences, Nowoursynowska 164, 02-787 Warsaw, Poland

**Keywords:** crossbreeding, reproductive performance, metabolic profile

## Abstract

Background: The high dairy production of Polish Holstein Friesian (PHF) cows determines high energy requirements in the early stages of lactation. Unfortunately, it is very often difficult to meet this demand through feedstuffs; therefore, homeostasis may be disturbed and metabolic diseases may occur, causing a majority of cows’ health problems. Breeders are, therefore, looking for alternatives to the PHF breed using crossbreeding. Methods: This experiment involved 30 PHF cows and 30 PHF × Swedish Red (SRB) crossbred hybrid cows, divided into two age groups, <2 years and >2 years, at first calving. Milk and blood samples were collected at 35 ± 5 days postpartum for analysis. Data on reproductive performance were also analyzed. Results: This study revealed lower milk production for the crossbreds hybrid (27.44 kg compared to 32.08 kg), with a higher basic composition content than PHF cows (fat: 3.97% compared to 3.83%, protein: 3.53% compared to 3.27%). The heifers of the crossbreds hybrid reached sexual maturity earlier but did not affect the lower age at first calving. Dividing the cows into age categories provided a more detailed perspective of the impact of genotypic differences on reproductive and metabolic profiles in PHF and PHF × SRB cattle. The findings highlight the importance of considering age-specific effects when assessing the performance and health of dairy cattle with diverse genotypes. Conclusions: The choice between PHF and PHF × SRB should depend on the specific goals and priorities of the cattle farming operation. Factors such as overall milk yield requirements, market demands, reproductive management strategies, and health considerations should be carefully evaluated to determine the most suitable breed for a given farming context.

## 1. Introduction

High animal performance is often a factor contributing to stress, leading to health problems. The most challenging period for dairy cows is undoubtedly the transitional period, which includes the final phase of pregnancy, calving, the onset of lactation, and the increase in productivity up to the peak of lactation, occurring at around the 100th day post-calving. During this time, dynamic changes in the body’s homeostasis can lead to the development of inflammatory conditions and a decrease in immunosuppressive abilities with hormonal origins, increasing the likelihood of metabolic, mammary gland, and reproductive system disorders [1,2,3,4,5]. During the transition period, metabolic disorders are by far the most important group of diseases that occur [3].

During this period, animals have the highest nutrient demand and require a properly balanced ration. Due to the sudden increase in the demand for energy and nutrients related to the initiation of colostrum and milk synthesis (fat, protein, and fatty acids), as well as the stress arising at this time, dairy cows are unable to take in an adequate energy supply with the normal ration. This results in a negative energy balance (NEB). Therefore, animals use the fat reserves accumulated in their body, turning them into energy and ketone bodies, causing the most common metabolic disorder: ketosis [2,5,6,7,8]. According to Gordon et al. [9], ketosis can occur in as many as 80% of all cows in individual herds. As reported by Duffield [10], clinical ketosis is diagnosed in 2 to 15% of all cows in the first two months of lactation, while McArt et al. [11] indicate that in the first week of lactation, 75% of high-yielding cows are diagnosed with subclinical ketosis, suggesting that this problem is a significant challenge for farmers. Among the changes observed by breeders occurring during the onset of ketosis in females are weight loss and a reduced condition. However, according to Horst et al. [4], weight loss immediately after parturition is a normal phenomenon in female mammals.

The loss of weight is related to the mobilization of adipose tissue to stimulate lactation, which is why many farmers do not pay much attention to the changes in the body weight of cows after parturition. Nevertheless, the prolongation of this state or rapid weight loss leads to the formation of stress in the body and, as a result, inflammation, which can cause the onset of ketosis and, subsequently, the onset of other diseases. Hence, an important aspect in the prevention of metabolic disease control is a timely diagnosis [12,13]. To detect their occurrence, a test is used to determine the level of ketone bodies, which are the intermediate metabolites of mobilized fat. Ketone bodies are formed during ketogenesis occurring in the liver, where the mitochondrial β-oxidation of long-chain fatty acids (FAs) occurs [6,14]. Ketone bodies include non-esterified fatty acids (NEFAs), β-hydroxybutyric acid (BHBA) acetone, and acetoacetic acid (in the form of acetoacetate anion) [15]. The levels of NEFA and BHBA increase during the course of the disease, while the levels of cholesterol and glucose, which play a key role in milk synthesis, decrease [14,16].

The diagnosis of metabolic disorders uses the determination of the concentration of ketone bodies in blood, urine, or milk. In the blood, the content of parameters such as NEFA, BHBA, or glucose is usually determined [17]. According to Serrehno et al. [17], analyses of urine and milk indicate the onset of a condition with a two-day delay compared to a blood test. In addition to ketone bodies, the determination of selected FAs, as well as whey proteins of milk, can be used in diagnosis [2,5,6,7,8].

Unfortunately, the occurrence of a negative energy balance during the transition period is very often the beginning of the problems farmers encounter with dairy cows. With the onset of NEB in cows, there are reproductive problems such as short, silent, or missing estrus, making it difficult to choose the right time for cows to mate. In addition, the pregnancy often does not develop even if fertilization occurs or there is a miscarriage at an early stage of embryonic development. All of this affects the extension of the postpartum downtime, increasing the number of semen straws used for successful insemination, and extending the service, the inter-pregnancy, and the inter-calving period, which will automatically decrease the milk yield [1,18,19,20].

Crossbred hybrids with an increased value of functional traits may prove to be an alternative to keeping high-yielding Holstein Friesian (HF) cows [21,22,23,24]. Crossbreeding is a well-known, appreciated, and often-used method of pairing individuals of two breeds into a parental pair, which is widely used in poultry production [25], pig breeding [26,27], and beef cattle [28]. The main advantage of this method is that it affects the improvement of less-heritable traits due to the possibility of the heterosis effect, defined as the exuberance of hybrids [27]. Crossbreeding between breeds positively influences many traits, encouraging the vigor of newborn offspring, health, body development, growth rate, feed utilization, productivity, and fertility [29]. According to Solarczyk et al. [30], crossbreeding dairy cattle with beef cattle has a beneficial effect on the bioactive molecule content of meat. There are also reports in the literature on crossbreeding dairy cattle in which it has been shown that functional traits such as fertility, health, and longevity are improved [29,31,32,33], as well as the content of bioactive milk fractions [34]. As reported by Sørensen et al. [31], due to low reproduction rates, there is growing interest in this method in many developed countries due to potential economic gain and possible consumer interest in products from hybrids.

To reduce the high cost of animal maintenance, studies have also been undertaken to determine the optimal age at the first calving (AFC) of dairy cows because the rearing of heifers for herd renovation has a significant influence on production costs. It is widely believed that ideally, the AFC should fall between 23 and 24 months of age [35,36,37], as a result of which cows achieve optimal results in milk and breeding performance.

The objective of this study was to assess the influence of genetic factors on key physiological traits in PHF cattle and PHF × SRB crossbreds. Particular attention was given to the effects of age at first calving, with cows categorized into two groups: those calving before 24 months and those calving at or after 24 months of age. The analysis focused on evaluating milk production, reproductive parameters, metabolic profiles, and fatty acid composition in relation to genotype. The results provide valuable insights into the genetic determinants of these traits and offer implications for optimizing breeding programs and herd management practices in dairy production.

## 2. Materials and Methods

### 2.1. Animals and Sampling

This study was conducted at the experimental dairy farm situated on the premises of the Warsaw University of Life Sciences (WULS) in Warsaw, Poland. This facility housed approximately 350 cows in a free-stall housing system, boasting an average lactation yield exceeding 10,000 kg of milk. Within the framework of this investigation, a meticulously selected group of 60 primiparous cows underwent thorough analysis, leading to their categorization into two distinct groups. The experimental group comprised 30 crossbreds identified as Polish Holstein Friesian × Swedish Red (PHF × SRB) cows, while the control group encompassed 30 purebred Polish Holstein Friesian (PHF) cows. The primiparous cows selected for the experiment were required to be in optimal health. Inclusion criteria specified the absence of locomotor disorders, with a particular emphasis on excluding any cases of hoof inflammation. Additionally, cows with a history of mastitis or related complications were excluded from the study. This study aimed to provide a detailed analysis of the age at first calving in PHF and PHF × SRB crossbreds cattle by categorizing the cows into two age groups: those less than 2 years old (<2 years; PHF—23.3 months, PHF × SRB—23.2 months) and those 2 years or older (>2 years; PHF—25.9 months, PHF × SRB—24.8 months). The sample comprised 15 PHF cows in the <2 years category, 15 PHF cows in the >2 years category, 15 crossbred cows in the <2 years category, and 15 crossbred cows in the >2 years category, ensuring equal representation of both breeds within each age class.

The dietary regimen was formulated based on the recommendations provided by the INRA system [38]. Administered ad libitum, the diet consisted of a total mixed ration (TMR) including maize silage (12.00 kg/d DM), alfalfa silage (4.20 kg/d DM), corn silage (2.10 kg/d DM), soybean meal (2.80 kg/d DM), pasture ground chalk (0.20 kg/d DM), salt (0.05 kg/d DM), rapeseed meal (1.80 kg/d DM), and magnesium oxide (0.06 kg/d DM). Notable nutritional parameters pertaining to the TMR included total kg of DM (23.10), daily intake (19.90 kg), net energy lactation (1.75 Mcal/kg), average milk production (37.02 kg), unit of milk production balance (3.45%), protein digested in the small intestine when rumen-fermentable nitrogen was limiting (2.51), and protein digested in the small intestine when rumen-fermentable energy was limiting (2.23).

Milk and blood were sampled at 35 ± 5 days postpartum. Milk and blood were collected from all 60 cows taking part in the experiment. Individual milk samples, each measuring 250 mL, were obtained during both morning and evening milking sessions. Subsequently, these samples were diligently preserved in sterile containers and expeditiously transported to WULS’s Milk Testing Laboratory for in-depth compositional analysis. In parallel, blood samples of 10 mL each were drawn from the jugular vein using specialized tubes (Vacuette, Essen, Germany). Post-collection, the blood samples underwent centrifugation at 1800× *g* and 4 °C for a duration of 15 min. The ensuing supernatant was promptly conveyed to WULS’s Veterinary Centre for a comprehensive suite of analyses.

Reproductive data were obtained from breeding records and included the following information: date of birth, date of first insemination, date of successful insemination, date of first calving, date of first service post-calving, date of successful service post-calving, the number of semen doses required for successful conception, and the date of subsequent calvings. Based on these records, the following reproductive parameters were calculated: AFI (age at first insemination), PI (pregnancy index), SP (service period), GL (gestation length), AFC (age at first calving), PPD (postpartum downtime), IP (inter-pregnancy interval), and PBC (calving interval). The service period (SP) was calculated as the interval between the first insemination and successful conception, with a value of 1 assigned in cases where conception occurred at the first estrus, in accordance with Kuczaj’s methodology [32]. The pregnancy index (PI) was calculated based on the number of semen straws used to achieve successful conception.

### 2.2. Chemical Analyses

The assessment of basic milk parameters, specifically fat and protein content, was conducted employing an automated infrared analysis methodology facilitated by a Milkoscan FT 120 analyzer (Foss Electric, Hillerød, Denmark).

The trans-esterification method outlined in EN ISO 12966-2:2017 [39] was employed for the methylation of FAs. The identification of individual FAs within crude fat samples was undertaken using an Agilent 7890A GC system (Agilent, Waldbronn, Germany), following the methodology established by Puppel et al. [40] The identification process was substantiated through the utilization of pure methyl ester standards, including FAME Mix RM–6 (Lot LB 68242), Supelco 37 Comp. FAME Mix (Lot LB 68887), Methyl linoleate (Lot 094K1497), and CLA Conjugated (9Z, 11E) (Lot BCBV3726), all sourced from Supelco (Bellefonte, PA, USA).

Quantitative glucose, protein, creatine, and GGTP analyses were conducted utilizing a BS800M biochemical analyzer (PZ Cormay, Warsaw, Poland) positioned within the Veterinary Centre of WULS. The reagents used for analysis with A-800 GLUCOSE (PZ Cormay, Warsaw, Poland) include enzymatic reagents such as glucose-6-phosphate dehydrogenase and NADP+. For A-800 GGT (PZ Cormay, Warsaw, Poland), gamma-glutamyl substrate, specifically gamma-glutamyl-p-nitroanilide, and a buffer were utilized. In the case of A-800 CREA ENZYMATIC (PZ Cormay, Warsaw, Poland), enzymes including creatinase and a buffer were employed. For A-800 TOTAL PROTEIN (PZ Cormay, Warsaw, Poland), copper was used with the biuret method, along with a buffer to stabilize pH.

### 2.3. Statistical Analysis

For the statistical analysis, an ANOVA was employed using the least-squares method to compare group means facilitated by the PS IMAGO PRO 7.0 software [41]. Prior to conducting an ANOVA, key assumptions were tested:

1. Normality: The Shapiro–Wilk test was applied to assess whether the data followed a normal distribution. All variables yielded *p*-values greater than 0.05, confirming that normality was not violated.
2. Homogeneity of Variance: Levene’s test for homogeneity of variances was conducted, ensuring that variances across groups were equal (*p* > 0.05). This indicates that the data met the assumption of homoscedasticity, essential for a valid ANOVA analysis.
3. Independence of Observations: Data collection procedures ensured that individual measurements were independent, as no repeat measures were taken from the same animals.

Given that all assumptions were satisfied, an ANOVA was deemed appropriate for comparing the differences between groups. Significant differences were identified using the F-statistic and post hoc comparisons were conducted where necessary to explore specific group differences.

These comprehensive checks ensured the validity and reliability of the statistical findings, providing confidence in the reported differences in reproductive parameters, metabolic profiles, and milk composition between the Polish Holstein Friesian (PHF) and PHF × Swedish Red (SRB) crossbred groups.

## 3. Results

### 3.1. Basic Composition of Milk

Table 1 presents the influence of cow genotype on the modulation of performance parameters pertaining to milk production as well as milk yield. Milk yield exhibited a significant 14.39% reduction in the PHF × SRB crossbreds relative to the PHF (*p*-value < 0.001). This divergence suggests nuanced genetic orchestration governing milk yield. Complementing this, the fat percentage subtly ascended by 3.47% in the PHF × SRB crossbreds compared to the PHF group (*p*-value = 0.045), revealing intricate lipid metabolism under genetic influences. Of paramount significance, the protein percentage escalated remarkably, by 7.83% within the PHF × SRB crossbreds group compared to the PHF (*p*-value < 0.001), signifying genetic modulation of protein synthesis. Casein content demonstrated a substantial elevation of 4.44% within the PHF × SRB crossbreds versus the PHF group (*p*-value < 0.001), denoting the genetic sway on specific milk protein fractions. Conversely, the fat-to-protein (F/P) ratio experienced a marginal decline of 2.88% in the PHF × SRB crossbreds relative to the PHF group (*p*-value = 0.098). Although statistically nonsignificant, this minor modulation underscores the intricate equilibrium between the lipid and protein pathways guided by genetic and environmental inputs (Table 1).

### 3.2. Reproductive Parameters

Significant differences emerged in the age at the first insemination. The PHF × SRB crossbreds exhibited an average age of 434.9 days, manifesting a substantial 4.32% reduction versus the PHF purebreds at 454.5 days (*p*-value < 0.001). This marked divergence underscores the genetic influence on the timeline of reproductive maturation. Conversely, the pregnancy index displayed a marginal 2.41% elevation in the PHF × SRB crossbreds compared to the PHF; however, this was insufficient for statistical significance (*p*-value = 0.465). This nuanced variation hints at the potential role of crossbreeding in reproductive success, although it does not conclusively establish a causal connection. Considering the service period, a pivotal metric of reproductive efficiency, PHF × SRB crossbreds exhibited a slight 4.31% extension (14.0 days) relative to the PHF group (13.4 days), albeit without statistical significance (*p*-value = 0.656). This marginal alteration suggests that while crossbreeding introduced genetic diversity, it may not substantially influence the temporal facet of reproductive cycles. The gestation length showcased a 0.21% elevation in PHF × SRB crossbreds (280.9 pregnancies per year) compared to the PHF (280.3 pregnancies per year). However, statistical insignificance (*p*-value = 0.200) implies that the effect of crossbreeding on gestation length remained subdued (Table 2).

During the postpartum downtime, both PHF × SRB (<2: 78.5 days, >2: 86.2 days) exhibited reductions versus the PHF (<2: 97.5 days, >2: 87.7 days) (*p*-value ≤ 0.001). This reduction underscores genetic influences on postpartum recovery, likely attributable to intricate genetic interactions shaping recovery rates. The pregnancy index, a pivotal determinant of reproductive efficiency, had PHF × SRB (<2: 2.27, >2: 1.54) demonstrating significantly lower indices relative to the PHF counterparts (<2: 2.09, >2: 2.43) (*p*-value ≤ 0.001), indicative of the intricate genetics governing reproductive prowess. The service period, a critical parameter of reproductive efficiency, exhibited similarity within age categories across both genetic groups (<2: 59.8 days, >2: 62.9 days for PHF; <2: 37.1 days, >2: 28.9 days for PHF × SRB) (*p*-value = 0.224), hinting at comparable temporal dynamics irrespective of genetic backgrounds. Likewise, the inter-pregnancy period, a hallmark of reproductive intervals, remained akin across age categories and genetic groups (<2: 157.3 days, >2: 150.6 days for PHF; <2: 115. 5 days, >2: 115.1 days for PHF × SRB) (*p*-value = 0.556), suggesting resilient temporal dynamics under diverse genetic contexts. Calving frequency, encapsulated within the period between calving, echoed consistency across all groups (*p*-value = 0.878), implying minimal influence of age categories or genetic backgrounds. Notably, the pregnancy period, mirroring gestational duration, had a significant reduction within crossbred animals (PHF × SRB) (<2: 283.3 days, >2: 278.2 days) versus their PHF counterparts (<2: 280.5 days, >2: 280.0 days) (*p*-value ≤ 0.001). This discrepancy implies that crossbreeding potentially alters gestational length through intricate genetic interplay, heralding new implications for herd management strategies (Table 3).

### 3.3. Metabolic Profile

Table 4 reports the influence of cow genotype and age first calving on the selected metabolic profile parameters. The NEFA is an important metabolite formed during the mobilization of adipose tissue for energy used to diagnose the occurrence of metabolic disorders. The examination of NEFA levels in PHF groups and PHF × SRB hybrids highlighted their clear differences. The interbreed hybrids (PHF × SRB) showed significantly lower NEFA levels compared to the corresponding PHF age groups. The NEFA value is approximately 70% higher in PHF cows than in hybrids.

BHBA is the most important indicator used to diagnose ketosis to study BHBA levels in the PHF and PHF × SRB hybrid groups. The hybrids showed lower BHBA levels in each group, while PHF cows that calved at over 2 years of age had very high BHBA levels, which may indicate a higher possibility of ketosis. Glucose, a vital metabolite reflecting metabolic equilibrium and cattle well-being, revealed breed-specific differences across distinct cattle groups. An examination of glucose levels within PHF and PHF × SRB groups highlighted notable distinctions. Crossbreds (PHF × SRB) consistently manifested significantly lower glucose levels compared to both age categories of PHF. The observed percentage differences of −1.81% for cattle with an age of first calving <2 years and a more pronounced −6.99% for those with an age of first calving >2 years accentuated the extent of these differences. The statistical significance of these findings (*p* < 0.001) underscored the role of genetic interactions and breed-specific factors in modulating metabolic processes, significantly influencing glucose homeostasis within the assessed cattle groups.

On transitioning to protein levels, pivotal indicators of physiological status, a comparison between the PHF and PHF × SRB groups revealed significant differences. Crossbreds cattle (PHF × SRB) consistently exhibited markedly lower protein levels across both age categories. The calculated percentage differences of −5.65% for cattle with an age of first calving <2 years and −2.97% for those with an age of first calving >2 years underscored the extent of these differences and the statistical significance (*p* = 0.016) highlighted the possible effects of genetic and breed-specific elements on protein metabolism, with potential implications for nutrient utilization within the studied cattle groups.

Albumins, crucial plasma proteins governing osmotic balance and molecular transport, revealed potential differences in nutrient utilization and overall health dynamics upon comparing the distinct cattle groups. A detailed analysis of albumin levels underscored significant differences between the PHF and PHF × SRB groups. Crossbreds cattle (PHF × SRB) consistently exhibited significantly higher albumin levels in both age categories than PHF cattle. The observed percentage differences of +6.42% for cattle with an age of first calving <2 years and a notable +13.75% for those with an age of first calving >2 years accentuated the potential influence of genetic and breed-specific components on nutritional metabolism, thereby affecting albumin dynamics and potential health implications within the studied cattle groups.

The analysis of creatinine levels, again, revealed notable differences between the PHF and PHF × SRB groups. Crossbreds cattle (PHF × SRB) consistently presented significantly higher creatinine levels within both age categories compared to PHF cattle. The observed percentage differences of +4.23% for cattle with an age of first calving <2 years and a notable +27.93% for those with an age of first calving >2 years underscored the potential influence of genetic and breed-specific factors on renal dynamics. The statistical significance of these differences (*p* < 0.001) pointed to the potential impact on creatinine levels and broader health implications within the studied cattle groups.

Gamma-glutamyl transferase (GGTP), a critical hepatic and biliary enzyme associated with liver health, exhibited significant differences within the PHF and PHF × SRB groups. Remarkably, crossbreds cattle (PHF × SRB) consistently displayed significantly lower GGTP levels in both age categories than PHF cattle. The observed percentage differences of −20.94% for cattle with an age of first calving <2 years and an even more substantial −35.47% for those with an age of first calving >2 years emphasized the significant nature of these differences. The statistical significance of these findings (*p* = 0.015) highlights the potential influence of genetic and breed-specific factors on hepatic function, potentially impacting GGTP levels and broader health implications within the studied cattle groups.

### 3.4. Fatty Acid Profile

Table 5 reports the influence of cow genotype and the age of first calving on the formation of selected FAs. PHF cattle calving at less than 2 years exhibited higher levels of C6:0 (1.868 g/100 g) in comparison to both the PHF × SRB groups (1.548 g/100 g for <2 and 1.600 g/100 g for >2). This difference was statistically significant (*p* = 0.001). Moving on to C10:0, there was a significant difference in levels between the PHF and PHF × SRB groups calving at 2 years or more, where the latter group had higher levels (2.745 g/100 g vs. 2.167 g/100 g), with a significant *p*-value of 0.024. A similar trend was observed in the case of C12:0, with PHF × SRB cattle calving at 2 years or more displaying higher levels (3.058 g/100 g) compared to PHF cattle (2.800 g/100 g), and this distinction was statistically significant (*p* = 0.001). Notably, while displaying higher levels in PHF × SRB cattle calving at 2 years or more (32.148 g/100 g) compared to PHF cattle (32.195 g/100 g), C16:0 did not exhibit statistical significance (*p* = 0.107), suggesting a more subtle difference. Moving to C18:0, no significant differences were observed between the PHF and PHF × SRB groups for either age category, with *p* = 0.587. However, C20:0 showed a substantial distinction, especially in cattle calving at less than 2 years. The PHF × SRB cattle in this category displayed significantly lower levels (0.072 g/100 g) compared to PHF cattle (0.243 g/100 g), as indicated by a *p*-value of <0.001. Two distinct conjugated linoleic acid isomers, CLA c9, tr11, and CLA tr10, c12, also exhibited significant differences. Both of these isomers were lower in the PHF × SRB groups compared to PHF cattle, irrespective of the age category, with *p*-values of <0.001 and 0.059, respectively. Lastly, C22:0, a metabolite with a relatively low presence, was significantly different between the groups, particularly in cattle calving at 2 years or more (*p* = 0.006).

## 4. Discussion

Adopting crossbreeding strategies in livestock production has sparked analytical interest due to the perceived advantages of crossbred animals over their parental breeds. Notably, research by Mäki-Tanila [42] has highlighted the improved robustness and economic efficiency associated with crossbreeding. This notion is further reinforced by the endorsements of Hansen [43] and Kalm [44], adding weight to the viability of crossbreeding as a pragmatic approach in livestock management. In Poland, the crossbreeding of dairy cattle has been quite popular for several years, and hybrids account for about 7% of the active population of animals [45].

### 4.1. Basic Composition of Milk

Milk production and the basic composition of milk are important aspects of cattle breeding, which, for many years, has been the primary breeding objective affecting the profit of dairy farming. Interesting information was provided by a study by Heins et al. [46] in which it was reported that HF cows have the highest productivity, followed by Holstein Friesian × Scandinavian Red (HF × SR) hybrids, then Holstein Friesian × Montbeliarde (HF × MO) hybrids, and the lowest in Holstein Friesian × Normande (HF × NO) hybrids. Most of the available studies provide information on milk yield for a full 305-day lactation, with the majority showing that the milk yield of HF × SR hybrids is lower than that of purebred HF cows [32,47,48,49,50], while Heins et al. [48] and Hazel et al. [51], in their studies, indicated a higher lifetime yield for HF × SR hybrids than purebred HF cows. Curiously enough, Benak et al. [52] confirmed this in the context of specific hybrids from the SR group of breeds, which are HF × NRF hybrids, and similar observations were made by Pytlewski et al. [53], whereas Ezra et al. [54] indicated that Holstein Friesian × Norwegian Red (HF × NRF) hybrids also have lower lactation performance. The confirmation of milk yield can be found in daily milk production. Our study showed a 14.39% reduction in milk yield within PHF × SRB crossbreds compared to PHF, indicating the genetic modulation of milk yield in HF and SRB breeds [55]. Similar lower milk yields with HF × SR were confirmed by Malchiodi et al. [56], Piccardi et al. [57], Saha et al. [58], Solarczyk et al. [59], Saha et al. [60], and Piazza et al. [61] in their studies. Interestingly, as in the case of higher productivity, higher daily milk production is observed in HF × NRF hybrids than in purebred HF cows, as indicated not only by the previously cited items but also in the study by Puppel et al. [34]. In the same study, the results of Holstein Friesian × Danish Red (HF × RDM) hybrids were also analyzed, where their productivity was lower than that of purebred HF cows. This is quite an interesting observation considering that in the improvement of the SR breed group, all the breeds included the Danish Red, Finnish Ayrshire, Norwegian Red, and Swedish Red breeds [62].

The study by Heins et al. [46] provided valuable insights into the variations in fat plus protein production among crossbred cows, showcasing distinct trends across different crossbred combinations. The HO × NO hybrids exhibited a significant 8.6% reduction in fat plus protein production compared to pure Holsteins, while the HF × MO crossbreds displayed a 3.8% decrease. In contrast, the HF × SR crossbreds showed a minor 2.2% reduction without statistical significance. Similarly, a 3.47% increase in fat percentage within PHF × SRB crossbreds signifies genetic nuances in lipid metabolism. Importantly, a remarkable 7.83% elevation in protein percentage within PHF × SRB crossbreds highlights genetic control over protein synthesis pathways, and a 4.44% rise in casein content (*p*-value ≤ 0.001) underscores genetic influence on specific milk proteins. The higher production of fat and protein in the milk of hybrids is confirmed by the available studies, which is certainly related to genetic determinants and lower milk production, which has a lower milk dilution effect [32,34,49,58,59,63]. Interesting results were obtained by Benak et al. [52], where the content of both fat and protein in the milk of HF × NRF hybrids was higher despite higher daily production than for the HF breed, while in the case of the results of Pytlewski et al. [53], the hybrids had a lower fat and protein content, which may confirm the dilution effect of the milk components. The higher casein content in the milk of HF × SR hybrids was similarly confirmed in our own study by Maurmayr et al. [64] and Puppel et al. [34].

### 4.2. Reproductive Parameters

The emphasis on increased production in cows frequently aligns with the detrimental effects on their health, fertility, and lifespan. Extensive investigation into the complex interplay of genetics governing production and functional traits like fertility and vulnerability to health concerns has resulted in diverse outcomes and incongruities across studies and reviews [65,66]. This inherent trade-off highlights the intricate complexities of simultaneously enhancing a range of traits in cattle breeding endeavors. Significantly, dairy cows with lower yields demonstrate a distinct advantage over their high-yielding counterparts, particularly concerning disease resistance. This distinction is particularly evident in aspects like udder health, fertility, longevity, and metabolic disorders. By comprehending these genetic antagonisms and trade-offs stemming from the selection for intense production, we acquired critical insights into the multifaceted task of enhancing livestock performance across diverse agricultural contexts [66,67]. These insights hold implications for refining breeding methodologies and management strategies with the aim of bolstering the sustainability and well-being of dairy cattle populations [66,68,69]. A significant reduction of 4.32% in the age of first insemination was demonstrated in PHF × SRB crossbreds compared to PHF purebreds. Similar values were obtained by Malchiodi et al. [70] in their studies with HF and HR × SR hybrids. Underlining the genetic influence on reproductive maturation timelines, according to Hutchison et al. [64], is a positive response to breeding programs and herd management because it increases profits. Due to the achievement of early sexual maturity and similar efficiency of the insemination procedure in PHF × SRB hybrids with purebred PHF heifers, the age at first calving (AFC) in the hybrids was also lower, at 729.9 days against 747.7 for the PHF breed. Taylor et al. [71] indicated that cows calving at <2 years of age exhibit longer lives and higher lifetime production. As indicated by Hutchison et al. [72], an earlier age of first insemination and consequently earlier AFC is related to better growth and fertility characteristics. As Berglund [73] points out, a huge influence on fertility is linked to breed and breeding goals. A decrease in the value of reproductive traits is mainly seen in the HF breed, even in animals from Scandinavia, where reproductive traits have been on the breeding program for 50 years, while in the Scandinavian Red breed group, the value of reproductive traits has remained constant for many years. Better fertility and a shorter parturition interval in purebred SRB cows compared to HF cows were also shown by Buckley et al. [74], Clasen et al. [75], Andree O’Hara et al. [55], and Bieber et al. [76] in their studies, confirming the higher value of reproductive traits in the SRB breed than the HF breed. During postpartum downtime, both age categories of PHF × SRB exhibited reductions in comparison to PHF, illustrating genetic influences on postpartum recovery. Similar results were obtained by Pipino et al. [77]. This underscores the intricate genetic and physiological interactions shaping recovery rates. As Hazel et al. [78] point out, metabolic disorders are diagnosed less frequently in HF × SR hybrids, and the costs related to treating these animals are lower than those related to treating HF cows. The pregnancy index, a vital determinant of reproductive efficiency, was significantly lower in both age categories of PHF × SRB compared to their PHF counterparts. Similar observations were obtained by Malchiodi et al. [70], Hazel et al. [51], and Pipino et al. [77]. Remarkably, the pregnancy period, reflecting gestational duration, exhibited a significant reduction within crossbred animals (PHF × SRB) compared to their PHF counterparts. This suggests that crossbreeding potentially alters gestational length through intricate genetic interactions, which has implications for herd management strategies. According to Pereira et al. [79], GL should be determined by the breed of the calf. In the case of this experiment, primiparous PHF × SRB hybrids were covered with MO breed bull semen, and the pregnancy of females calved for the first time at less than two years of age lasted 283.3 days; this was consistent with previous reports on the effect of the MO breed on gestation length [49,79].

### 4.3. Metabolic Profile

Differences in metabolic parameters between purebred HF cows and their hybrids with SR primarily arise from distinct metabolic pathways influencing energy mobilization and fat metabolism during lactation. The process of lipid mobilization in dairy cows is critical during both the transition period and early lactation. In purebred HF cows, rapid fat mobilization can lead to increased levels of NEFA and BHBA, indicative of a higher risk of ketosis [80]. In contrast, SRB hybrids may exhibit more regulated lipid mobilization, potentially due to genetic differences that enhance FA oxidation. This may involve more active pathways mediated by peroxisome proliferator-activated receptors (PPARs), resulting in lower NEFA and BHBA concentrations during early lactation [81]. Our research confirms that there is a greater mobilization of fats at the beginning of lactation in PHF cows than in PHF × SRB hybrids, as indicated by higher levels of ketone bodies in PHF cows. The BHBA levels in calving cows over 2 years of age were particularly worrying: BHBA PHF <2; 0.725, >2; 1.111 to PHF × SRB <2; 0.695, >2; 0.711 mmol/L. According to Hazel et al. [78], HF × SR hybrids do not show metabolic problems as often as HF cows, which is related to the SR breed’s influence on these animals’ metabolism. Differences in metabolism between HF and SRB cows were proven by Ntallaris et al. [82], where a lower NEFA value was shown in SRB cows than in HF cows. The metabolism of body fat is also influenced by the amount of fat stored, according to Ospina et al. [83]. Cows that enter the reproductive period later tend to accumulate more fat, which may, at the same time, result in a greater mobilization of spare matter in the postpartum period.

Glucose metabolism is responsible for the formation of energy and hormones; being highly dependent on insulin is another key factor differentiating these breeds. Insulin sensitivity may vary between HF and SRB cows, with SRB cows likely exhibiting improved insulin responsiveness. This enhanced sensitivity may facilitate greater glucose uptake and utilization during lactation. The process of gluconeogenesis, particularly from propionate derived from fiber digestion, may be more efficient in SRB cows, leading to elevated blood glucose levels compared to HF cows. The regulation of gluconeogenic enzymes such as phosphoenolpyruvate carboxykinase (PEPCK) and glucose-6-phosphatase could be more effective in SRB hybrids, promoting stable glucose concentrations. In our own research, low glucose levels were observed in cows calving at >2 years of age in both PHF breeds and PHF × SRB hybrids; however, as reported by Mohammed et al. [84], the accepted values are at an appropriate level. According to Ntallaris et al. [82], blood glucose content in HF cows is lower than in SRB cows, which could account for the influence of breed on this parameter; however, based on our research, we can conclude that fat mobilization and the formation of ketone bodies affect gluconeogenesis quite strongly, during which glucose is formed [80]. The higher blood glucose content in cows calving at less than 2 years of age may also indicate a lower demand for energy due to a smaller body frame [85].

Additionally, variations in blood protein levels, specifically total protein and albumin, may indicate differences in immune responses and inflammation. Elevated protein levels in HF cows often correlate with inflammatory states during early lactation. This inflammation is mediated by pro-inflammatory cytokines, including interleukin-6 (IL-6) and tumor necrosis factor-alpha (TNF-α), influencing protein metabolism. In contrast, SRB hybrids may demonstrate a more robust immune response, potentially due to genetic traits that enhance immune function and reduce inflammatory responses [86].

Creatinine levels, as a marker of muscle metabolism, may also differ between these breeds [87]. In HF cows, the increased mobilization of protein reserves to meet energy demands may lead to lower creatinine concentrations, reflecting greater protein catabolism. Conversely, SRB hybrids may maintain muscle mass more effectively, resulting in higher creatinine levels.

Liver enzyme activity such as gamma-glutamyltransferase (GGTP) is indicative of hepatic health and metabolic stress. HF cows often exhibit elevated GGTP levels, particularly those calving at older ages, which may signify liver stress and fat accumulation. Excessive fat mobilization during early lactation can overload the liver’s capacity for fat oxidation, resulting in fatty liver syndrome and increased GGTP [8,88]. In contrast, SRB hybrids may demonstrate superior hepatic function and lipid metabolism, potentially linked to an enhanced expression of genes involved in fatty acid oxidation and liver regeneration. In conclusion, the observed differences between purebred HF cows and SRB hybrids stem from a complex interplay of genetic, biochemical, and physiological factors influencing lipid and glucose metabolism, protein catabolism, immune function, and hepatic health.

### 4.4. Fatty Acid Profile

The observed differences in milk FA profiles between PHF cows and PHF × SRB hybrids can be understood through the complex interaction of genetic, metabolic, and physiological factors that influence lipid metabolism. These differences can be attributed to the distinct metabolic pathways involved in de novo FA synthesis in the mammary gland and the mobilization of FAs from adipose tissue, alongside the regulation of these processes by genetic and metabolic factors such as breed-specific characteristics, metabolic health (e.g., ketosis), and the stage of lactation [89].

Breed-related genetic differences, particularly between PHF and SRB cows, contribute significantly to the variation in milk FA composition. Genetic selection in dairy breeds like HF has historically focused on high milk yield, which can indirectly influence lipid metabolism. In contrast, breeds like SRB are typically selected for their robustness, including greater resistance to metabolic disorders. This divergence in breeding objectives leads to differences in the regulation of key enzymes involved in FA synthesis pathways such as acetyl-CoA carboxylase (ACC) and FA synthase (FAS), which are essential for the de novo synthesis of short- and medium-chain FAs in the mammary gland [90].

The de novo synthesis of FAs (up to C16:0) occurs primarily in the mammary gland from precursors such as acetate and BHBA, which are produced during rumen fermentation. The differences in the levels of caproic acid, capric acid, and lauric acid in our study could reflect breed-specific differences in the activity of these metabolic pathways. For example, Poulsen et al. [91] demonstrated that SRB cows tend to have lower levels of certain medium-chain FAs compared to Holstein Friesians. This may be linked to lower the activity of enzymes like FAS in SRB cows, resulting in a reduced synthesis of C6:0 and C10:0, as observed in our study. Conversely, higher levels of de novo FAs in PHF cows can be associated with greater FAS and ACC activity, possibly due to genetic selection for high milk yield.

The metabolic state of the cow, particularly during early lactation when cows are in a negative energy balance, significantly impacts the mobilization of FAs from adipose tissue. In this phase, cows often exhibit elevated levels of NEFAs in the bloodstream due to the mobilization of stored triglycerides, which are then transported to the liver for β-oxidation or directed to the mammary gland for incorporation into milk fat. The mobilization of long-chain FAs such as stearic acid and arachidic acid primarily originates from adipose tissue rather than de novo synthesis [6].

In cows experiencing a negative energy balance or metabolic stress such as ketosis, the excessive mobilization of adipose reserves leads to increased concentrations of long-chain FAs in milk. This can explain the higher levels of C18:0 and C20:4 in PHF cows, which are often more prone to metabolic disorders like ketosis due to their high milk production demands. As suggested by Puppel et al. [6], higher concentrations of BHBA in cows with subclinical ketosis are associated with an altered FA metabolism, potentially leading to increased levels of mobilized FAs, including C18:0. Our findings that PHF cows had higher levels of these long-chain FAs support this notion, indicating that HF may be more susceptible to mobilizing body fat during early lactation.

Metabolic disorders such as ketosis significantly affect lipid metabolism and consequently the FA composition of milk. In cases of ketosis, elevated levels of BHBA, a product of incomplete FA oxidation in the liver, indicate an impaired energy metabolism. During ketosis, cows are unable to meet their energy requirements through dietary intake alone, resulting in the excessive breakdown of adipose tissue triglycerides and increased NEFA levels in circulation. These NEFAs are then incorporated into milk fat, altering the fatty acid profile, and increasing the levels of C18:0 and other long-chain FAs [2] in particular.

Additionally, ketosis is associated with shifts in the synthesis of conjugated linoleic acid isomers in milk. The CLA is primarily formed in the rumen through the biohydrogenation of linoleic acid, and its synthesis is modulated by rumen microbial activity and the metabolic state of the cow. CLA9 and CLA10, two bioactive isomers, have been identified as potential biomarkers for metabolic health [5]. In our study, the higher levels of CLA9 in PHF × SRB hybrids calving before 2 years of age and lower levels of CLA10 in hybrids calving after 2 years suggest that these isomers may be reflective of the metabolic status and possibly the incidence of ketosis. As noted by Puppel et al. [6], higher CLA concentrations, particularly CLA10, have been associated with cows in better metabolic health, while lower levels may indicate subclinical metabolic disturbances.

The variation in palmitic acid levels observed in our study, with higher levels in cows calving after 2 years of age, reflects this shift. Palmitic acid, synthesized both de novo and through mobilized lipids, is one of the most abundant FAs in milk. As lactation advances, healthy cows typically exhibit higher C16:0 concentrations, as noted by Puppel et al. [6], which is indicative of improved metabolic stability and efficient fat synthesis. The higher levels of C16:0 in older cows in our study may reflect better energy management and metabolic health in these animals, as younger cows, particularly those calving before 2 years of age, often face greater metabolic stress during lactation.

The differences in milk FA composition between PHF cows and PHF × SRB hybrids arise from the interplay of breed-specific genetic factors, metabolic health, and lactation dynamics. These factors influence key metabolic pathways, including the de novo synthesis of FAs in the mammary gland and the mobilization of adipose-derived FAs. Genetic differences between PHF and SRB cows affect the activity of enzymes such as ACC and FAS, leading to variations in medium-chain FAs, while metabolic disorders like ketosis alter lipid mobilization, increasing the concentration of long-chain FAs. The stage of lactation further modulates these processes, with de novo synthesis predominating as cows return to a positive energy balance. These findings underscore the importance of understanding the metabolic and genetic factors influencing milk composition, as they have implications for both dairy production efficiency and animal health management.

## 5. Conclusions

In summary, the crossbred PHF × SRB cows demonstrated notable advantages in milk composition, reproductive efficiency, and postpartum recovery time. While PHF cows exhibited a higher total milk yield compared to PHF × SRB hybrids, the latter group displayed enhanced levels of fat, protein, and casein in their milk, which may better align with specific market demands and processing requirements. The reproductive performance of the PHF × SRB hybrids was characterized by earlier sexual maturation compared to purebred PHF cows, indicating an improvement in reproductive efficiency. Although the pregnancy index was marginally elevated in PHF × SRB hybrids, the service period for PHF cows was longer; however, this extension did not reach statistical significance.

In addition to reproductive metrics, the metabolic profiles of the two breeds provided further insight into their physiological adaptations. Distinct differences in metabolic parameters—such as glucose, protein, albumin, creatinine, and GGTP levels—were observed, reflecting the unique physiological dynamics of each breed. For instance, crossbreds exhibited consistently lower glucose and protein levels compared to PHF cows, which may indicate different energy mobilization and metabolic efficiencies. The lower glucose levels in PHF × SRB hybrids could suggest a more efficient utilization of available energy, particularly in younger cows, while variations in albumin and creatinine levels may reflect differing health statuses and muscle condition between the breeds.

An analysis of the age at first calving, categorized into those calving at less than 2 years and those at 2 years or older, revealed that PHF × SRB hybrids experienced a significant reduction in milk yield relative to PHF across both age categories. Nevertheless, there was a marked increase in fat, protein, and casein content in the milk of the crossbreds, indicating a genetic influence on these components. The genotypic impact was further highlighted by the timing of first insemination, with PHF × SRB hybrids reaching sexual maturity earlier, particularly among those calving before 2 years of age. Although the pregnancy index showed a slight increase in crossbreds, the service period was also extended, although this change was not statistically significant in either age group.

The division into age categories facilitated a comprehensive understanding of how genotypic differences affect first-calving age, milk production, reproductive metrics, and metabolic traits in both PHF and PHF × SRB cattle. These findings underscore the necessity of considering age-specific effects when evaluating the performance and health of dairy cattle with diverse genetic backgrounds. Ultimately, the choice between PHF and PHF × SRB should be guided by the specific objectives and priorities of the cattle farming operation. A careful assessment of factors such as overall milk yield, market demands, reproductive management strategies, and health considerations is essential to determine the most suitable breed for a given agricultural context.

## Figures and Tables

**Table 1:** Influence of cow genotype on the formation of performance parameters of milk and milk yields.

	PHF (n = 30)		PHF × SRB (n = 30)		*p*-Value
	LSM	SEM	LSM	SEM	
Milk yield [kg]	32.08	0.292	27.44	0.297	<0.001
Fat [%]	3.83	0.046	3.97	0.047	0.045
Protein [%]	3.27	0.016	3.53	0.016	<0.001
Casein [%]	2.77	0.011	2.89	0.011	<0.001
F/P	1.17	0.015	1.14	0.015	0.098

PHF—Polish Holstein Fresian; PHF × SRB—Polish Holstein Fresian × Swedish Red; LSM—least-square means; SEM—standard error of LSM; F/P—fat/protein.

**Table 2:** The influence of cow genotype on the formation of reproductive parameters.

	PHF (n = 30)		PHF × SRB (n = 30)		*p*-Value
	LSM	SEM	LSM	SEM	
AFI [days]	452.5	1.40	434.9	1.42	<0.001
PI [in units]	1.44	0.033	1.48	0.034	0.465
SP [days]	13.4	0.95	14.00	0.96	0.656
GL [days]	280.3	0.32	280.9	0.32	0.200

PHF—Polish Holstein Fresian; PHF × SRB—Polish Holstein Fresian × Swedish Red; LSM—least-square means; SEM—standard error of LSM; AFI—the age of the first insemination; PI—pregnancy index; SP—service period; GL—gestation length.

**Table 3:** Influence of cow genotype and age of first calving on the formation of reproductive parameters.

AFC	PHF				PHF × SRB				*p*-Value
	LSM	SEM	LSM	SEM	LSM	SEM	LSM	SEM	
	<2 (n = 15)		>2 (n = 15)		<2 (n = 15)		>2 (n = 15)		
AFC [days]	708.3	1.67	787.1	1.48	705.0	1.67	754.4	1.54	<0.001
PPD [days]	97.5	2.23	87.7	1.97	78.5	2.23	86.2	2.05	<0.001
PI [in units]	2.09	0.082	2.43	0.073	2.27	0.082	1.54	0.076	<0.001
SP [days]	59.8	4.87	62.9	4.31	37.1	4.87	28.9	4.48	0.224
IP [days]	157.3	5.49	150.6	4.87	115.5	5.49	115.1	5.05	0.556
PBC [days]	437.7	5.38	430.6	4.77	398.8	5.38	393.3	4.95	0.878
GL [days]	280.5	0.47	280.0	0.41	283.3	0.47	278.2	0.43	<0.001

PHF—Polish Holstein Fresian; PHF × SRB—Polish Holstein Fresian × Swedish Red; LSM—least-square means; SEM—standard error of LSM, AFC—the age of the first calving; PPD—period of postpartum downtime; PI—pregnancy index; SP—service period; IP—inter-pregnancy period; PBC—the period between calving; GL—gestation length.

**Table 4:** The influence of cow genotype and age of first calving on the selected metabolic profile parameters.

Age	PHF				PHF × SRB				*p*-Value
	LSM	SEM	LSM	SEM	LSM	SEM	LSM	SEM	
	<2 (n = 15)		>2 (n = 15)		<2 (n = 15)		>2 (n = 15)		
NEFA [mmol/L]	0.405	0.042	0.420	0.104	0.281	0.042	0.282	0.038	<0.001
BHBA [mmol/L]	0.725	0.038	1.111	0.034	0.695	0.038	0.711	0.035	<0.001
Glucose [mg/dL]	65.234	0.817	61.812	0.724	64.061	0.817	60.559	0.751	<0.001
Protein [g/L]	70.488	1.204	66.640	1.067	66.725	1.204	64.690	1.107	0.016
Albumins [g/L]	35.999	0.583	35.356	0.517	38.453	0.583	40.306	0.537	0.008
Creatynine [mg/dL]	0.970	0.022	0.939	0.019	1.016	0.022	1.198	0.020	<0.001
GGTP [U/L]	19.572	2.652	24.506	1.215	16.427	2.652	15.853	1.638	0.015

PHF—Polish Holstein Fresian; PHF × SRB—Polish Holstein Fresian × Swedish Red; LSM—least-square means; SEM—standard error of LSM; NEFA—non-esterified fatty acids; BHBA—β-hydroxybutyric acid; GGTP—gamma-glutamyl transferase.

**Table 5:** The influence of cow genotype and age of first calving on the formation of selected fatty acids.

Age	PHF				PHF × SRB				*p*-Value
	LSM	SEM	LSM	SEM	LSM	SEM	LSM	SEM	
	<2 (n = 15)		>2 (n = 15)		<2 (n = 15)		>2 (n = 15)		
C6:0 [g/100g fat]	1.868	0.045	1.457	0.040	1.548	0.045	1.600	0.042	< 0.001
C10:0 [g/100g fat]	1.983	0.087	2.167	0.077	2.929	0.087	2.745	0.080	0.024
C12:0 [g/100g fat]	2.247	0.090	2.800	0.080	2.909	0.090	3.058	0.083	0.001
C16:0 [g/100g fat]	27.655	0.388	32.195	0.344	29.238	0.388	32.148	0.357	0.107
C18:0 [g/100g fat]	13.102	0.231	11.983	0.205	10.236	0.231	9.802	0.213	0.587
C20:0 [g/100g fat]	0.243	0.010	0.153	0.009	0.072	0.010	0.103	0.009	< 0.001
CLA9 [g/100g fat]	0.480	0.012	0.437	0.011	0.518	0.012	0.469	0.011	< 0.001
CLA10 [g/100g fat]	0.038	0.003	0.030	0.003	0.034	0.03	0.018	0.003	0.059
C22:0 [g/100g fat]	0.011	0.006	0.063	0.005	0.020	0.006	0.048	0.005	0.006

PHF—Polish Holstein Fresian; PHF × SRB—Polish Holstein Fresian × Swedish Red; LSM—least-square means; SEM—standard error of LSM; CLA9—CLA *cis-9*, *trans 11*; CLA10—CLA *trans-10*, *cis-12*.

## Data Availability

All data generated or analyzed during the study are included in this published article. The datasets used and/or analyzed in the current study are available from the corresponding author on reasonable request.
